# Aging Biology of Bone-to-Tendon Healing and the Epigenetic Clock: A Biological-Age Readout of Rotator Cuff Healing Capacity

**DOI:** 10.3390/biomedicines14091980

**Published:** 2026-09-02

**Authors:** Jong Pil Yoon, Sung-Jin Park, Dong-Hyun Kim, Chul-Hyun Cho, Yuki Yoshida, Hailey Nam, Seok Won Chung

**Affiliations:** 1 Department of Orthopedic Surgery, Kyungpook National University Hospital, Daegu 41944, Republic of Korea; altjp1@gmail.com (J.P.Y.); miniself@hanmail.net (S.-J.P.); kdh8110@hanmail.net (D.-H.K.); 2 Department of Orthopedic Surgery, Dongsan Hospital, Keimyung University School of Medicine, Daegu 42601, Republic of Korea; oscho5362@dsmc.or.kr; 3 Department of Orthopaedic Surgery, Fussa Hospital, Tokyo 197-8511, Japan; yuutree.yysy@gmail.com; 4 Department of Orthopedic Surgery, Konkuk University Medical Center, Seoul 05030, Republic of Korea; hailey.nam8@gmail.com

**Keywords:** rotator cuff, bone-to-tendon healing, enthesis, epigenetic clock, biological age, tendon–bone interface, regenerative medicine

## Abstract

The “unexplained failure” of rotator cuff repair is multifactorial, but its structural endpoint is anatomically consistent, i.e., the failure of the tendon-to-bone interface (enthesis) to heal. The native enthesis is a four-zone fibrocartilaginous gradient that does not regenerate but heals as a mechanically inferior fibrovascular scar, so the outcome of repair hinges on the interface’s healing capacity—which chronological age predicts poorly. This review organizes the aging biology governing bone-to-tendon healing capacity into eight domains: progenitor competence, cellular senescence and the SASP, immune aging, extracellular-matrix and collagen aging via advanced glycation end-product cross-linking, footprint angiogenesis, morphogen signaling, mechanotransduction, and bone quality. We then precisely define the DNA-methylation epigenetic clock—a continuous value produced by weighted CpG methylation, with defined units, reproducibility, and effect sizes—and propose it as a candidate quantitative readout of these domains; whether or not it truly integrates them into a single biologically meaningful measure at the enthesis is a hypothesis of this review, not an established mechanism. In 1087 twins, epigenetic age acceleration predicted fracture and osteoporosis risk, with hazard ratios of 1.29–3.17 per standard deviation; moreover, aging is tissue-specific, so the enthesis may run ahead of blood. Critically, the clock provides a single axis on which current regenerative-medicine strategies—stem cells, exosomes, immunomodulation, biomimetic gradient scaffolds, growth factors, senolytics, and epigenetic reprogramming—can be systematically categorized by how far each shifts biological age toward a healing-competent state; partial reprogramming, which rewinds the clock directly, shows that the clock is simultaneously the readout and the therapeutic target. We integrate this into a “hidden biological age of bone-to-tendon healing”, explicitly stating that this remains an unvalidated hypothesis requiring prospective validation.

## 1. Introduction

Over the past decade, aging research has undergone a fundamental shift: aging is no longer understood solely through chronological age but can be quantified as an individual’s biological age obtained from DNA-methylation patterns. Since Horvath and Hannum introduced epigenetic clocks that predict age from the methylation of hundreds of CpG sites in 2013 [1,2]—followed by second-generation clocks trained on mortality and morbidity (PhenoAge, GrimAge) [3,4] and the third-generation pace-of-aging clock (DunedinPACE) [5]—the concept has become one of the most actively studied aging biomarkers in major journals such as *Nature*, *Science*, and *Cell*. Epigenetic alteration is now established as a hallmark of aging [6], and by measuring not “how old” but “how aged” a person is, these clocks are shifting the medical paradigm from chronological to biological age.

This shift is more than conceptually attractive because epigenetic age has repeatedly outperformed chronological age as a predictor across clinical domains. Epigenetic age acceleration independently predicts all-cause mortality [7,8] and is consistently associated with cancer incidence and survival [8,9], cardiovascular disease [8], frailty [10], and neurodegenerative change including Alzheimer’s disease [11]; metabolic syndrome and type 2 diabetes risk are likewise linked to epigenetic aging. Systematic review and meta-analysis confirm that these associations reproduce across cohorts [12], and in one German cohort, a methylation age running five years ahead of chronological age significantly raised the risk of all-cause, cancer, and cardiovascular mortality [8]. The paradigm has further extended to the skeleton: in 1087 twins, epigenetic age acceleration strongly predicted fracture and osteoporosis risk [13]. In short, the epigenetic clock is a highly promising next-generation biomarker that quantitatively supports the intuition that aging is the common substrate of disease.

What, then, does this paradigm mean for predicting rotator cuff repair prognosis? The variables we currently use to gauge healing prognosis—age and the presence of diabetes, obesity, smoking, metabolic disease, and osteoporosis—are in fact surrogates that do not directly reflect a tissue’s regenerative capacity. Two patients with the same chronological age of 65 may have entirely different biological aging states, and two diabetic patients may carry vastly different metabolic aging burdens; even patient-reported satisfaction after arthroscopic repair has been reported to differ by age group [14]. These traditional predictors are thus low-precision, indirect proxies that do not directly measure tendon healing potential, regenerative capacity, inflammatory response, or tissue-remodeling capacity. The epigenetic clock, being grounded in DNA methylation, reflects the actual biological aging process significantly more directly and may therefore capture these healing determinants with greater accuracy. The central message of this review is not simply that “an epigenetic clock could be measured”, but that it could serve as a next-generation biomarker capable of replacing crude clinical predictors and bringing a paradigm shift to rotator cuff healing prediction.

Rotator cuff tears are common shoulder disorders affecting close to half of the population older than 50 years, and arthroscopic repair is the standard of care [15,16]. Despite advances in technique and construct design, structural failure after repair remains frequent, and the structural integrity of the repair has repeatedly emerged as the key determinant of clinical outcome [17]. Muscle atrophy and fatty infiltration are well-established, independent prognostic factors for both structural and functional outcome: these conditions cause poor recovery after repair and correlate with poor function [18,19]. Yet the anatomical event of structural failure itself, i.e., the loss of continuity between the repaired tendon and bone, occurs at the tendon-to-bone interface (enthesis). Muscle quality and interface healing are therefore interdependent determinants of the same repair: a degenerated muscle imposes an unfavorable mechanical and biological environment on the healing interface, while a failed interface in turn perpetuates muscle degeneration. This review focuses on the enthesis because it is the site at which failure ultimately manifests and the substrate on which regenerative interventions act and not because muscle factors are secondary.

**Figure 1:**
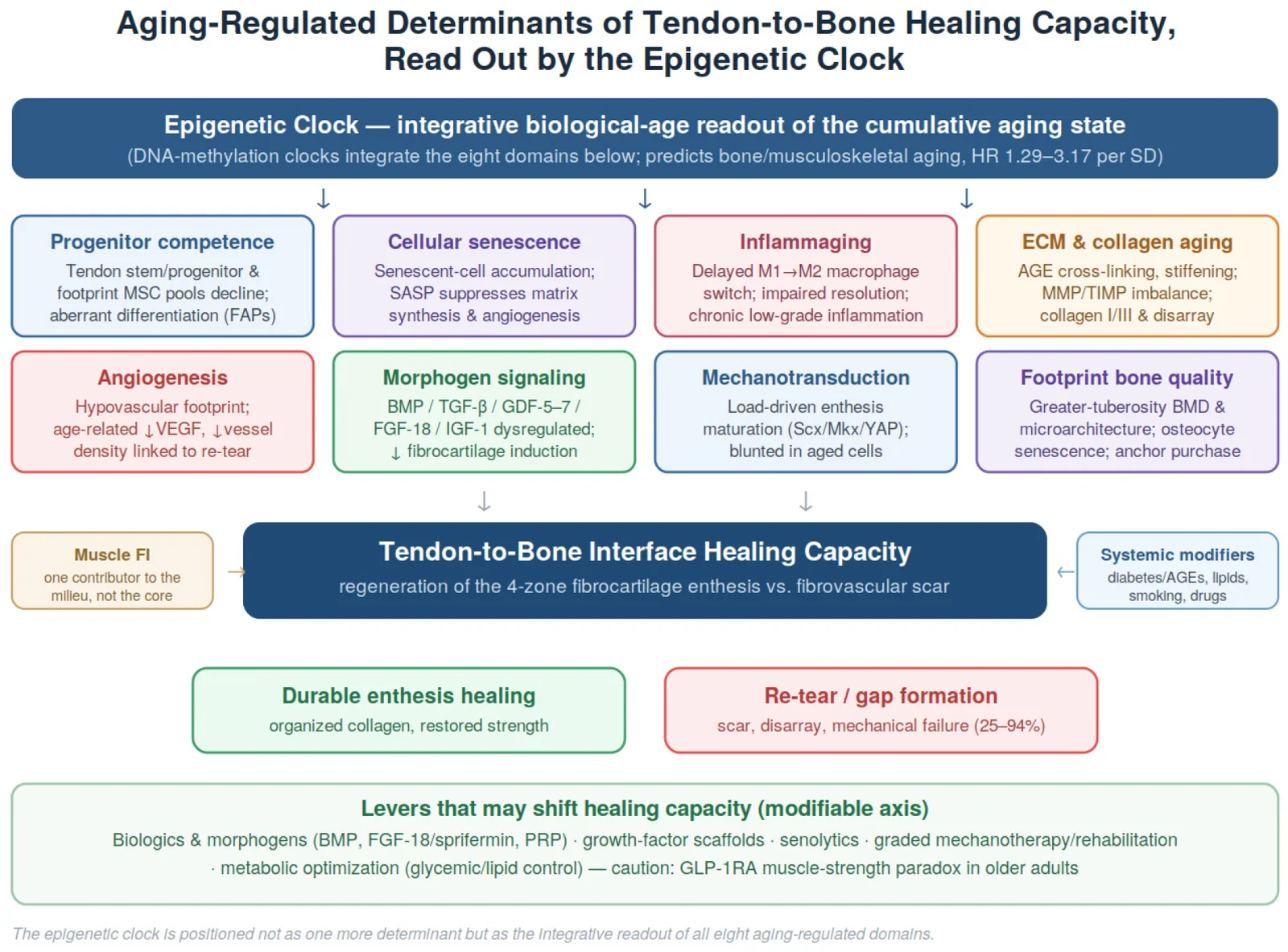
Tendon-to-bone interface healing capacity is placed at the center, surrounded by the eight aging-regulated determinant domains (progenitor competence, cellular senescence, inflammaging, ECM/AGEs, angiogenesis, morphogen signaling, mechanotransduction, and bone quality), with the epigenetic clock hypothesized as their integrative readout, and a set of modifiable levers. Muscle fatty infiltration and systemic metabolism, both established prognostic factors in their own right, are shown as codeterminants interacting with the interface milieu.

The essential outcome variable in rotator cuff repair is therefore how mature a healing the tendon-to-bone interface achieves, i.e., its healing capacity. Yet chronological age, the most convenient clinical surrogate for risk, predicts this healing capacity poorly. Among two patients who are both 70 years old, one interface may heal robustly while the other fails, with scar and gap formation. This review organizes the aging biology that produces this inter-individual difference into multiple domains and proposes the DNA-methylation-based epigenetic clock as a biological-age readout that integrates those domains (Figure 1).

We state at the outset that the evidence assembled here derives from other tissues and clinical conditions; no study has yet directly linked epigenetic age to rotator cuff healing, and the framework of this review is therefore an extrapolative hypothesis to be tested, not an established mechanism.

That evidence was identified by searching PubMed, Scopus, and Web of Science, supplemented by manual searches of reference lists and of recent issues of major shoulder and aging-biology journals, using combinations of the terms “rotator cuff”, “enthesis”, “tendon-to-bone (bone-to-tendon) healing”, “epigenetic clock”, “DNA methylation”, “biological age”, “epigenetic age acceleration”, “cellular senescence”, “inflammaging”, “partial reprogramming”, and “regenerative medicine”. For the aging-biology and epigenetic-clock literature, we prioritized randomized controlled trials, systematic reviews and meta-analyses, and landmark mechanistic studies; for the orthopedic literature, we prioritized preclinical and clinical studies directly addressing tendon-to-bone interface healing and validated prognostic factors of rotator cuff repair. Only English-language publications were included. This is a narrative review: no systematic protocol (e.g., PRISMA) was followed, article selection reflects the authors’ judgment of relevance to the proposed framework, and a potential for selection bias should accordingly be considered when interpreting the synthesis.

## 2. The Tendon-to-Bone Interface: The True Outcome Substrate

The native enthesis dissipates stress through a four-zone gradient of tendon, unmineralized fibrocartilage, mineralized fibrocartilage, and bone. This intricate architecture does not regenerate after injury; following repair it heals as a mechanically inferior fibrovascular scar. Consequently, the degree to which collagen alignment, density, and tensile strength are restored becomes the central histologic determinant of repair success. Indeed, a series of animal studies by the author’s group quantified bone-to-tendon interface healing by collagen I expression, the collagen alignment index, the Bonar score, and ultimate failure load and showed that these histologic and biomechanical indices sensitively reflect treatment effect [20,21]. The finding that a scaffold with spatially embedded growth factors promotes interface healing in a rabbit model of chronic rotator cuff tear supports the view that the structural completeness of the interface is an independent and modifiable prognostic factor [22].

It must be emphasized, however, that the healing of this interface does not occur in a biological vacuum: it is powerfully conditioned by well-established mechanical and structural parameters. Tear size and tendon retraction remain among the strongest predictors of structural failure [17,23]. Tendon quality [17,18,19], the repair construct and its time-zero fixation mechanics [16,24], and postoperative variables such as rehabilitation and patient compliance [16] all measurably influence whether the repaired tendon remains apposed to bone long enough for healing to proceed. These parameters and the aging biology reviewed below are best understood as two complementary axes of a single multifactorial model: the mechanical axis defines the load environment and the technical integrity of the repair, whereas the biological axis, comprised of the aging domains detailed in Section 3, determines the intrinsic capacity of the interface to heal within that environment. A small, minimally retracted tear repaired with an optimal construct may still fail in a biologically aged interface. Conversely, a biologically young interface cannot compensate for an overtensioned or mechanically insufficient repair. Accordingly, the biological-age framework proposed in this review is intended to complement, not replace, the established mechanical predictors, and any future predictive model incorporating an epigenetic clock will need to be validated against and ultimately integrated with these structural variables.

## 3. Aging-Regulated Determinants of Healing Capacity

The healing capacity of the tendon-to-bone interface is determined not by a single factor but by the sum of several aging domains (Figure 1, Table 1).

First is the competence of progenitor cells, comprising the cellular engine of healing. Reconstruction of the interface depends on the appropriate differentiation of tendon-derived progenitors, footprint marrow stromal cells, and fibro-adipogenic progenitors (FAPs); aging reduces the size and differentiation fidelity of these pools and diverts FAPs toward adipogenic and ectopic fates [25]. Second is cellular senescence and the senescence-associated secretory phenotype (SASP). As senescent cells accumulate, the SASP suppresses matrix synthesis and angiogenesis, and the observation that senolytic intervention restores bone aging and responsiveness to mechanical loading suggests that this axis is causal [26].

Third is immune aging (inflammaging). Tendon healing proceeds through inflammatory, proliferative, and remodeling phases, and these transitions are orchestrated by M1→M2 macrophage polarization, which inflammaging delays while impairing resolution; the observation that nerve injury worsens enthesis regeneration underscores the importance of the local microenvironment [27]. Fourth is the aging of the ECM and collagen. In aged tenocytes, MMP-2/9 activity rises, degrading the collagen matrix and disrupting its alignment [28], and more fundamentally, AGE cross-linking formed by non-enzymatic glycation stiffens collagen and impedes remodeling. This is the mechanism by which hyperglycemia and metabolic syndrome are linked to tendon-injury risk and by which diabetic animals show altered rotator cuff tissue [29,30].

The fifth and sixth domains—“supply and command”—are angiogenesis and morphogen signaling. The footprint is intrinsically hypovascular, and reduced rotator cuff vascularity is associated with tear and re-tear pathology [31]; aging further diminishes the VEGF response and vessel density. In parallel, morphogens such as BMP, TGF-β, GDF-5–7, FGF-18, and IGF-1 induce fibrocartilage formation, and the demonstration that recombinant FGF-18 (sprifermin) improves tendon-to-bone healing through chondrogenesis [32], together with the effect of growth-factor scaffolds [22], shows that this axis is a modifiable target. The seventh and eighth domains—“stimulus and anchorage”—are mechanotransduction and bone quality. Enthesis maturation depends on appropriate load (Scx/Mkx/YAP-TAZ signaling), and aged cells may show a blunted mechanoresponse that weakens the effect of rehabilitation [27]; moreover, anchor fixation and intraosseous tendon integration depend on greater-tuberosity bone quality, which osteocyte senescence and declining bone density erode, and which factors such as greater-tuberosity microfracture have been investigated to enhance [13,26,33]. Notably, the muscle-derived myokine irisin increases cortical bone mass, suggesting that muscle–bone crosstalk can influence footprint bone quality [34].

## 4. The Epigenetic Clock: A Quantitative Definition

An epigenetic clock must first be precisely defined. An epigenetic clock is not a vague “degree of aging” but a single continuous number produced by a weighted linear combination of DNA-methylation levels (β values, continuous from 0 to 1) at a defined set of CpG sites. Concretely, the methylation values of tens to hundreds of CpGs are fitted by penalized regression (for example, elastic net) against an aging-related outcome to output a biological age in years—or, for the third-generation DunedinPACE, a rate of aging in years per calendar year. Its measurement method (methylation array or sequencing), its units (years, or years per year), its reproducibility, and its effect sizes (hazard ratio per standard deviation) are all defined. In this sense the epigenetic clock is a biomarker that can be handled as a number rather than as a qualitative impression, and it is precisely this quantitative character that makes it eligible for integration into surgical decision making.

## 5. The Epigenetic Clock as a Hypothesized Integrative Readout

**Figure 2:**
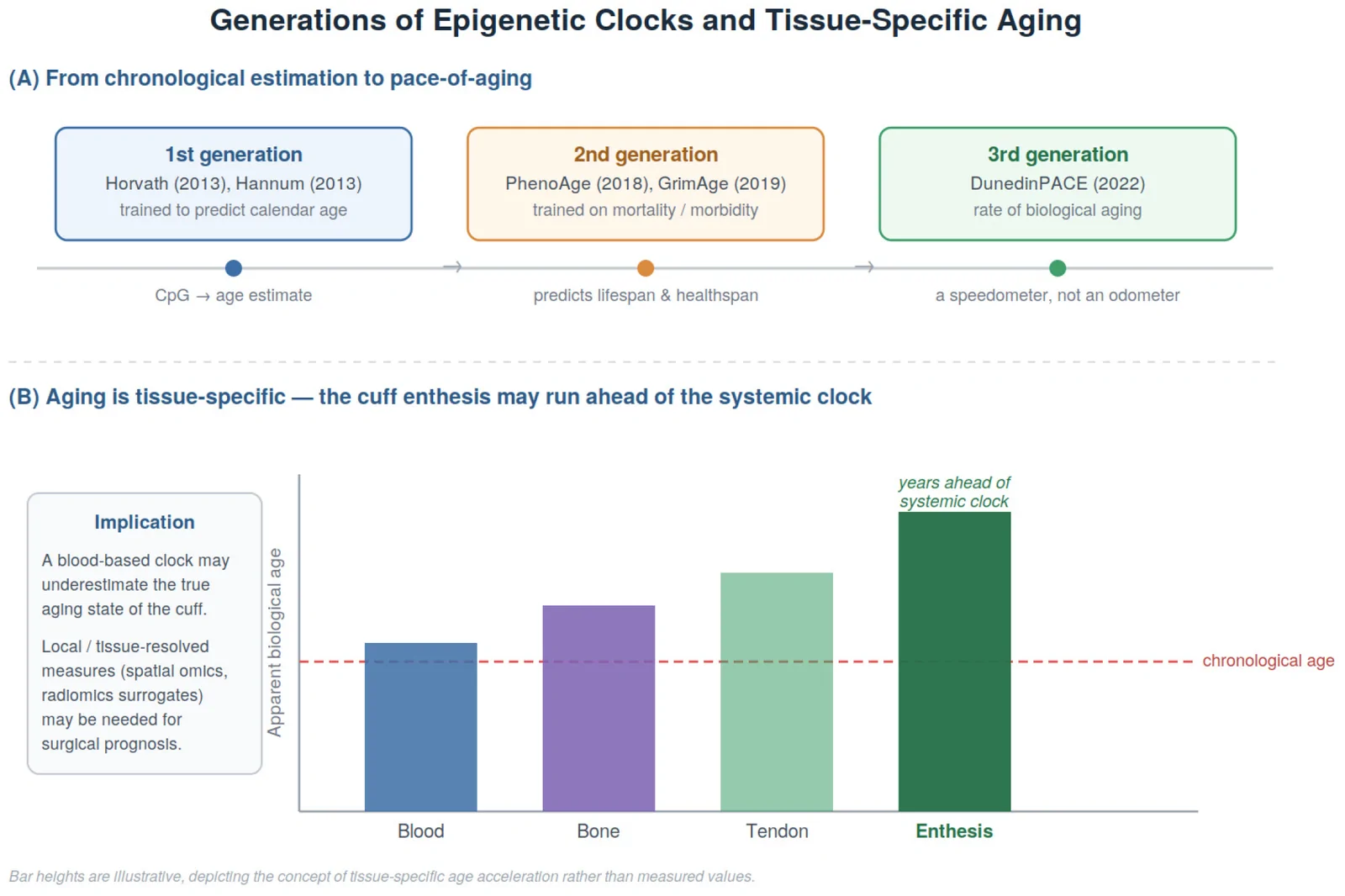
(**A**) Evolution of epigenetic clocks across generations [1,2,3,4,5]. (**B**) Tissue-specificity of aging. A blood-based clock may underestimate the true aging state of the rotator cuff enthesis, whose biological age may run ahead of the systemic clock. Bar heights are illustrative of the concept. The proposition in (**B**) that enthesis biological age runs ahead of that of the systemic clock is a hypothesis of this review; no direct measurement of enthesis methylation age currently exists.

Each of these eight domains can be measured and targeted, but assessing all of them separately is impractical in clinical care. The epigenetic clock is attractive because, in principle, it would not merely sum these domains as parallel factors but capture the cumulative aging state in a single biological age (their generational development and tissue-specificity are summarized in Table 2 and Figure 2). We emphasize, however, that this integrative capacity at the rotator cuff enthesis is a hypothesis of the present review: the evidence cited below demonstrates that epigenetic clocks predict bone-aging outcomes, not that they integrate the eight proposed domains into a single biologically meaningful measure for the enthesis, a hypothesis which has never been tested.

The orthopedic relevance of this integrative readout comes from a recent twin study. Analyzing 1087 twins, the study showed that epigenetic age acceleration is consistently associated with fracture and osteoporosis risk, most robustly for DunedinPACE and GrimAge, with hazard ratios of 1.29–3.17 per standard deviation, after genetic and environmental confounding were removed [13]. Furthermore, it is established that the pace of aging differs among tissues within the same individual [1]. Whether the rotator cuff enthesis in particular carries a more advanced epigenetic age than do other tissues, however, has never been directly measured; this proposition is an untested hypothesis of the present review, supported only by indirect plausibility from the chronic mechanical loading, hypovascularity, and degenerative burden of the footprint. If the hypothesis holds, a blood-based clock alone would risk underestimating the true aging of the enthesis, which argues for tissue-resolved measurements such as spatial transcriptomics or imaging-based surrogates [35], and direct methylation profiling of enthesis tissue is accordingly the first requirement of the research agenda in Section 10.

## 6. Modifiability of Healing Capacity

Healing capacity is not fixed and can be shifted upward by interventions across several categories (systematized on the clock axis in the next section); metabolic and nutritional optimization is one such axis. Preclinical work by the author’s group showed that ezetimibe/atorvastatin improves collagen alignment and tensile strength [21] and that a GLP-1 receptor agonist and metformin improve the muscle environment [36,37], while nutritional status, such as vitamin D level, has been linked to rotator cuff health and surgical results [38]. Yet, in older adults, GLP-1 receptor agonists may paradoxically accelerate handgrip-strength loss and sarcopenia [39], underscoring that heterogeneous effects across age and metabolic status must be weighed.

## 7. Categorizing Regenerative-Medicine Advances on the Clock

**Figure 3:**
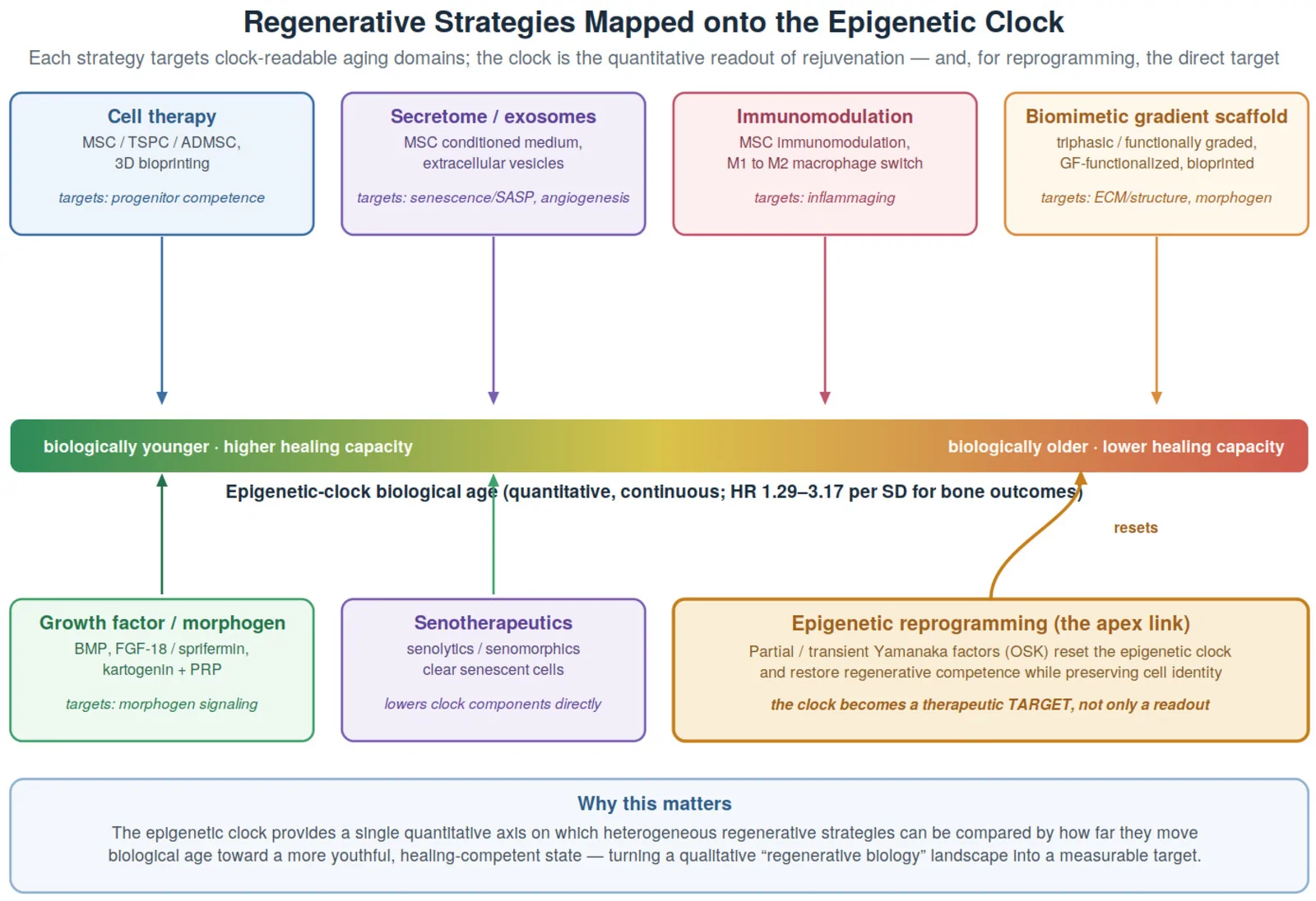
Mapping of heterogeneous regenerative-medicine strategies onto the aging domains read out by the epigenetic clock. Each strategy targets specific domains, and the clock becomes a common axis quantifying how far biological age is shifted toward a younger state. Partial reprogramming is the apex link that resets the clock directly, showing the clock to be simultaneously a readout and a therapeutic target.

Regenerative medicine for rotator cuff bone-to-tendon healing is among the most active research areas today, yet the use of stem cells, exosomes, immunomodulation, biomimetic scaffolds, growth factors, senolytics, and epigenetic reprogramming is reported in different languages, making integrated comparison difficult. This review proposes to categorize these advances systematically on a single quantitative axis—the aging domains that the epigenetic clock reads out (Figure 3, Table 3).

The key insight is that each regenerative strategy targets a specific aging domain and that the clock could serve as a common metric of how far a given strategy rejuvenates the tissue—contingent on the validation of the integrative hypothesis above. Cell-based strategies—implantation of mesenchymal stem cells (MSCs), tendon-derived progenitor cells (TSPCs), and adipose-derived stem cells (ADMSCs), and three-dimensional bioprinting with autologous ADMSCs—directly replenish the progenitor-competence domain [40]. Cell-free secretome strategies—MSC conditioned medium and exosomes (extracellular vesicles)—act on the senescence/SASP and angiogenesis domains to recondition the aged microenvironment [40]. Immunomodulatory strategies target the inflammaging domain through MSC immunomodulation and the M1→M2 macrophage switch [41]. Biomimetic gradient scaffolds and patches—triphasic or functionally graded constructs, growth-factor-embedded bioprinted patches, and acellular dermal matrix augmentation—address the ECM/structure domain and morphogen presentation simultaneously, and recent work has reported large-animal studies, biomechanical evaluations, and short-term clinical outcomes [20,22,23,24,42,43]. Growth-factor and morphogen strategies (BMP, FGF-18/sprifermin, kartogenin plus PRP) target the morphogen-signaling domain, while senolytics and senomorphics target the senescence/SASP domain and may directly lower the components of the clock [26,32].

At the apex of this categorization sits epigenetic reprogramming. Partial, transient expression of Yamanaka factors (OSK) rewinds the epigenetic clock while preserving cell identity, lowering biological age and restoring regenerative competence across multiple tissues, including skeletal muscle [44]. Here, the clock is no longer a passive readout but a therapeutic target in itself. At the same time, the fact that quantifying biological age as cells acquire younger states has emerged as a central challenge during reprogramming [45] paradoxically demonstrates why the clock is needed as a common metric of regenerative efficacy. In sum, the epigenetic clock renders heterogeneous regenerative strategies comparable on a single quantitative axis—the extent to which each shifts biological age toward a younger, healing-competent state—turning a once-qualitative landscape of regenerative biology into a measurable target.

## 8. Long-Term Clinical Scenarios: What the Epigenetic Clock Could Ultimately Enable

The epigenetic clock is a powerful and innovative concept because, beyond being a mere correlative marker, it has repeatedly demonstrated in top-tier journals that aging is a quantity that can be not only measured but manipulated. The key milestones fall into two strands. First, interventions actually move the clock. In an analysis of the CALERIE randomized controlled trial—the first well-controlled RCT in which an intervention slowed a methylation clock—the pace of aging measured by DunedinPACE was significantly slowed [46]; a small human study aimed at thymic regeneration (TRIIM) likewise observed a reversal of epigenetic age [47]. Second, reprogramming can “rewind” the clock: partial expression of Yamanaka factors ameliorated hallmarks of aging [48] and, in a separate report, restored vision after optic-nerve injury, while resetting the epigenetic age to a younger state [49]. The clock is thus not a passive marker but a causally manipulable quantity—an essential feature of a robust, mechanistic biomarker.

The concept is also rapidly industrializing. Cellular reprogramming is currently the most heavily funded idea in aging science, with companies such as Altos Labs (launched in 2022, with roughly US $3 billion), Retro Biosciences, and NewLimit competing in the field, and in 2026, a partial epigenetic reprogramming gene therapy (Life Biosciences’ ER-100) received US FDA clearance as the first-in-human trial in patients with vision loss [49]. In parallel, DNA-methylation-based biological-age tests are being commercialized, and epigenetic clocks are increasingly adopted as quantitative surrogate endpoints in interventional aging trials. This momentum matters for orthopedics because it suggests that standardized, scalable, quantitative biological-age measurement may become progressively accessible, although, as discussed in Section 9, substantial technical and validation barriers remain.

The boundary of clinical support must be drawn explicitly at this point. The evidence summarized above—an RCT slowing a methylation clock through caloric restriction [46], a small human study observing epigenetic-age reversal [47], and the regulatory clearance of a first-in-human reprogramming trial in vision loss—is entirely systemic or non-orthopedic [49]. No epigenetic-clock-based application of any kind—diagnostic, prognostic, or therapeutic—is currently supported by clinical evidence in rotator cuff care, and none of the concepts that follow, including “rejuvenate-then-repair”, molecular-age-timed surgery, aging-signature-matched biologics, and clock-guided reprogramming, has been tested in any musculoskeletal patient population. Everything beyond this point is hypothesis.

**Figure 4:**
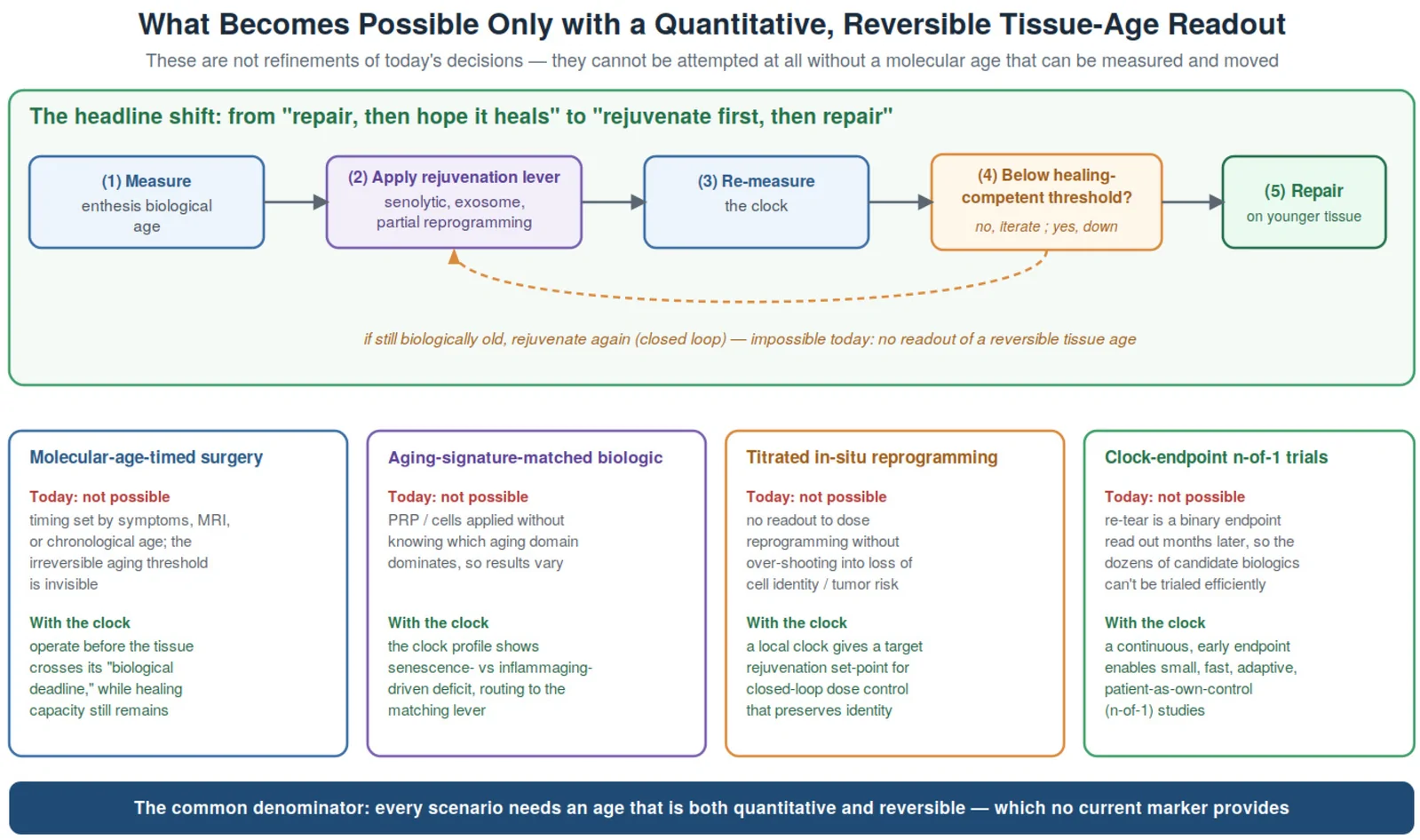
Long-term conceptual scenarios that would become possible only with a validated, quantitative, reversible tissue-age readout. The headline paradigm is a shift from “repair, then hope it heals” to a closed loop of “rejuvenate first, then repair”; each card contrasts what is not feasible today with what a validated clock could enable. All scenarios are hypothetical long-term goals, not current clinical options.

The conceptual point is that the clock would not merely make existing variables “a little more accurate”. Because it is an age that is both quantitative and, in principle, reversible, it could ultimately open surgical strategies that no current tool can support. We emphasize at the outset that none of the scenarios below is achievable using present technology or evidence: each presupposes a validated tendon/enthesis-specific clock, which does not yet exist, and all should therefore be read as long-term research goals—the conceptual endpoints of a research program—rather than immediate clinical perspectives. With this caveat, what follows are not near-term uses, such as risk stratification or rehabilitation tuning, but scenarios that could not even be formulated without such a clock (Figure 4, Table 4).

The most fundamental long-term shift would be from a surgery that “repairs and then waits for healing” to one that “makes the tissue younger first, then repairs”. Today, there is no way to measure whether a preoperative intervention (senolytics, exosomes, exercise, metabolic optimization) actually rejuvenated the interface, so this “rejuvenate-then-repair” strategy cannot even be formulated. With the clock, a closed loop becomes possible: measure tissue biological age → apply a rejuvenation lever → re-measure → repair only once the tissue has dropped below a healing-competent threshold. By the same logic, the timing of surgery is set by molecular age rather than in regards to symptoms or structure: the clock can signal a “biological deadline” so that surgery is performed before the interface crosses an irreversible aging threshold—while healing capacity still remains—a judgment that chronological age, MRI, and symptom-based timing cannot make in principle.

Second, which regenerative therapy a given patient should receive is decided by an “aging signature”. Today PRP or cells are applied without knowing which aging domain dominates, but a clock profile distinguishes a senescence-driven from an inflammaging-driven deficit and routes the patient to the matching lever—senolytic, anti-inflammatory, or morphogenic—drug selection grounded in aging biology. Third, if partial reprogramming—now entering early-phase trials in other organ systems—were ever applied locally, it could, in principle, be titrated: its chief danger is over-dedifferentiation (loss of identity, tumor risk), and a local enthesis clock that provides a target rejuvenation set-point enables closed-loop dose control that stops short of compromising identity. Fourth, measuring local vs. systemic age separately defines the target—if the enthesis alone is old while the body is young, a local biologic can be used instead of a systemic drug (spatial targeting). Finally, the clock completely changes the way in which rotator cuff biologics are studied: today, the binary, months-later re-tear endpoint makes it impossible to trial the dozens of candidate biologics efficiently, whereas a continuous, early clock endpoint shrinks sample sizes and enables adaptive, patient-as-own-control (n-of-1) studies. What these scenarios share is that none can even begin before a molecular-age readout that is both quantitative and reversible has been developed and validated for the enthesis.

## 9. Discussion: Toward a Hidden Biological Age of Bone-to-Tendon Healing

This evidence converges on a single integrative hypothesis: the prognosis of rotator cuff repair is determined by the healing capacity of the tendon-to-bone interface; that capacity is the sum of aging domains—progenitor competence, cellular senescence, inflammaging, ECM/AGEs, angiogenesis, morphogen signaling, mechanotransduction, and bone quality—and this cumulative aging state may be captured more accurately by an integrative biological-age measure such as an epigenetic clock than by chronological age. We tentatively term this the “hidden biological age of bone-to-tendon healing”.

This integrative metric, however, has not been validated in any prospective cohort of rotator cuff patients and remains an explicit hypothesis. The present evidence supports the belief that (1) epigenetic clocks predict bone aging, (2) bone-to-tendon healing capacity is composed of measurable phenotypes across multiple aging domains, and (3) those domains can be modulated by interventions of several kinds. It does not establish that these domains integrate into a single biological-age index that predicts repair outcome. This limitation—that the central construct is unvalidated—should be kept in view when interpreting the framework, and it defines the work that must follow.

Beyond this conceptual limitation, the practical barriers of current epigenetic-clock technology must also be weighed before any clinical application is contemplated. Assay standardization remains incomplete: methylation values are sensitive to batch effects, bisulfite-conversion efficiency, and normalization pipelines, and no clinically certified workflow for clock estimation yet exists in musculoskeletal care, while array-based genome-wide profiling still costs on the order of several hundred US dollars per sample, feasible for research cohorts but not for routine preoperative testing. Tissue access is likewise asymmetric to the clinical question: the enthesis can be sampled only intraoperatively, whereas the decisions this framework ultimately envisions—patient selection, surgical timing, and preoperative rejuvenation—are preoperative, so blood-based clocks or imaging surrogates, which may underestimate the true aging state of the enthesis given the tissue-specificity of aging discussed above, would first need to be validated as intermediaries. Reproducibility and comparability add further constraints: technical noise at individual CpG probes can shift first-generation clock estimates by several years between technical replicates, a variability that principal-component-based reformulations reduce but do not eliminate [50], and probe content differs among the Illumina 450K, EPIC, and EPICv2 arrays and sequencing-based approaches, so clocks trained on one platform do not transfer to another without recalibration. Most fundamental to this review, no epigenetic clock has yet been trained or validated on tendon or enthesis tissue; existing clocks derive from blood or multi-tissue panels that do not include tendon tissue, and the marked cellular heterogeneity of the fibrocartilaginous transition zone means that bulk methylation signals conflate shifts in cell composition with intrinsic cellular aging. The precedent of a dedicated skeletal-muscle clock, which outperformed the pan-tissue clock within muscle [51], indicates that a tendon/enthesis-specific clock is achievable in principle, but it must first be constructed and validated before any of the applications discussed in Section 8 can proceed. Taken together, these constraints reinforce that the clinical scenarios proposed in this review are long-term research goals rather than near-term clinical tools.

## 10. Future Directions

**Figure 5:**
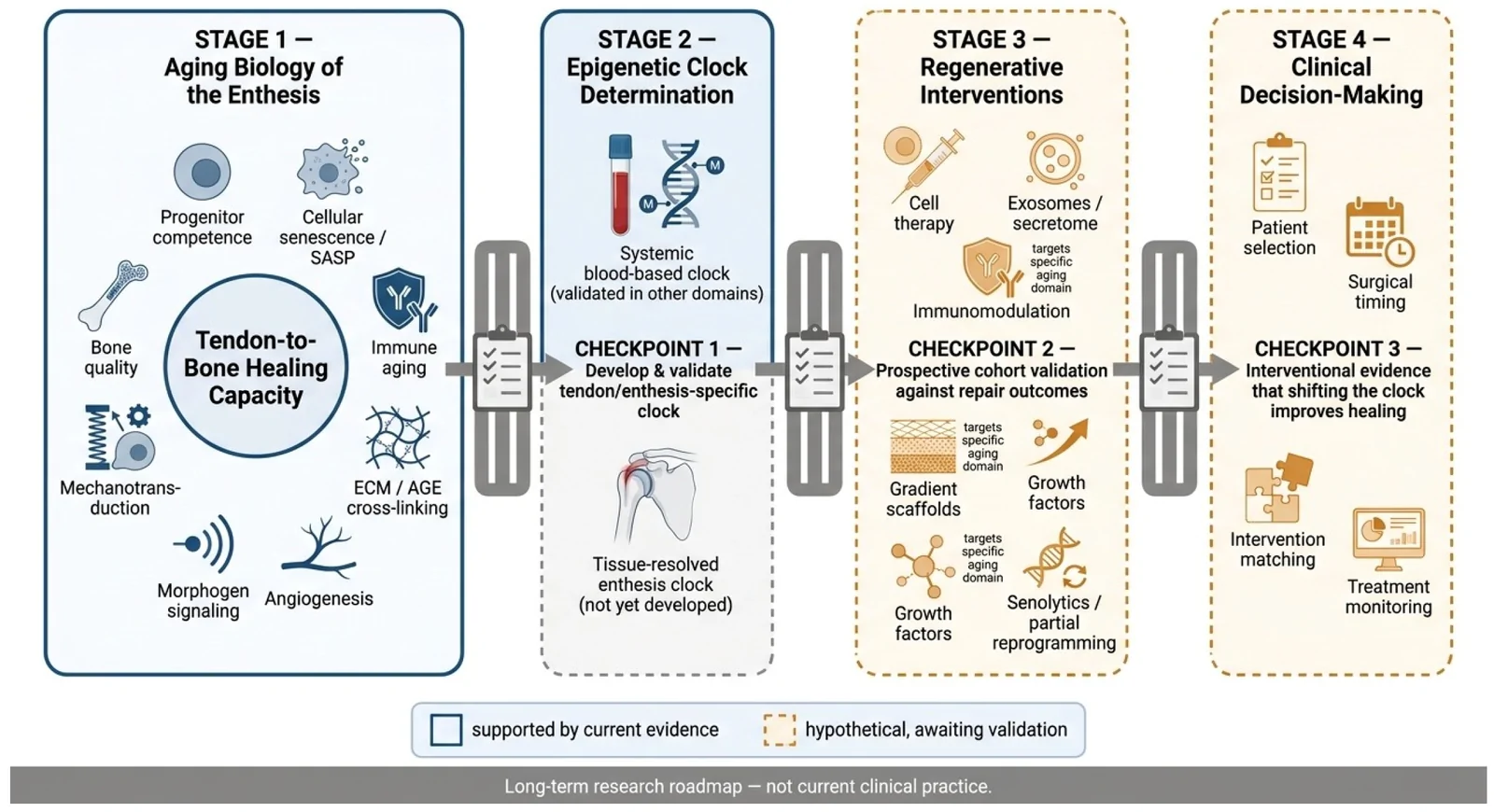
Proposed translational path of the hidden-biological-age framework. Stage 1, biological mechanisms of aging (the eight domains governing tendon-to-bone healing capacity); Stage 2, epigenetic clock determination (systemic blood-based and, ultimately, tissue-resolved enthesis-specific measurement); Stage 3, regenerative interventions categorized by the aging domains they target; Stage 4, possible clinical decision making (patient selection, surgical timing, intervention matching, and treatment monitoring). Arrows between stages indicate the validation checkpoints required for each transition: development and validation of a tendon/enthesis-specific clock (1→2), prospective cohort validation against repair outcomes (2→3), and interventional evidence that shifting the clock improves healing (3→4). Solid outlines denote currently evidence-supported elements; dashed outlines denote hypothetical elements awaiting validation. The entire downstream path remains a long-term research goal.

The translational path from the aging biology reviewed here to eventual clinical decision making is summarized in Figure 5, which also marks the validation checkpoints that must be crossed at each transition. Moving the hidden-biological-age framework from hypothesis to validated tool will require progress along three lines. First, prospective cohorts undergoing rotator cuff repair should measure preoperative epigenetic age (systemic and, where feasible, tissue-resolved), tissue aging markers, and imaging surrogates simultaneously, and then follow interface healing and re-tear in order to test whether these domains integrate into a single predictive biological-age index [13,35]. Second, tissue-resolved readouts—spatial transcriptomics and imaging-based surrogates—are needed because a blood clock may underestimate enthesis aging [35]. Third, analytic and regulatory infrastructure—standardized methylation assays, multi-omic integration, survival modeling, and the artificial-intelligence and machine-learning pipelines now entering shoulder practice [52]—will be required to translate a continuous biological-age readout into bedside decisions. Throughout, the GLP-1 receptor agonist paradox in older adults [39] is a reminder that rejuvenation levers can act heterogeneously and must be validated against the very clock they aim to move.

## 11. Conclusions

The “unexplained failure” of rotator cuff repair is multifactorial, and its structural endpoint is the failure of bone-to-tendon healing—a capacity governed by the biology of multiple aging domains and conditioned by established muscle-based and mechanical prognostic factors. The epigenetic clock, as a candidate readout hypothesized to integrate these domains into a single biological age, holds the potential to capture the inter-individual variation in aging missed by chronological age. Healing capacity can be shifted upward by growth factors, scaffolds, senolytics, mechanotherapy, and metabolic optimization, though the paradox in older adults must be considered. Binding this evidence into a “hidden biological age of bone-to-tendon healing” is an attractive hypothesis, but prospective validation is the prerequisite. We hope this review can serve as a starting point for adding “bone-to-tendon healing” alongside established muscle-based predictors as an outcome variable in rotator cuff prognosis research, with “biological age”, alongside “calendar age”, as a measurement variable.

## Figures and Tables

**Table 1:** Aging-regulated determinants of tendon-to-bone interface healing capacity.

Aging Domain	Role in Bone-to-Tendon Healing	Effect of Aging	Evidence
Progenitor competence (TSPC/MSC/FAP)	Cellular reconstruction of the interface via differentiation	Reduced pool/differentiation; FAP adipogenic drift	[25]
Cellular senescence/SASP	Supports matrix synthesis and angiogenesis	Senescent-cell accumulation; suppressed synthesis	[26]
Immune aging	M1→M2 switch drives healing phases	Delayed switch; impaired resolution	[27]
ECM/collagen (AGEs)	Collagen alignment, density, tensile strength	↑MMP activity; AGE cross-linking stiffening	[28,29,30]
Angiogenesis	Perfusion of the hypovascular footprint	↓VEGF/vessel density → re-tear risk	[31]
Morphogen signaling	BMP/TGF-β/FGF-18 induce fibrocartilage	Dysregulated signaling and responsiveness	[22,32]
Mechanotransduction	Load-driven enthesis maturation	Blunted mechanoresponse in aged cells	[27]
Footprint bone quality	Anchor purchase; intraosseous tendon fixation	↓Tuberosity BMD; osteocyte senescence	[13,26,33]

**Table 2:** Generations of major epigenetic clocks.

Generation	Representative Clock (Year)	Training Target	Relevance to Musculoskeletal Aging
First	Horvath, Hannum (2013) [1,2]	Chronological age	Established multi-tissue feasibility; provides a baseline
Second	PhenoAge (2018), GrimAge (2019) [3,4]	Mortality/morbidity (clinical biomarkers, plasma proteins)	Stronger links to fracture and osteoporosis
Third	DunedinPACE (2022) [5]	Pace of aging (longitudinal rate)	Largest effect sizes for bone outcomes in the twin study

**Table 3:** Systematic categorization of regenerative-medicine strategies: targeted aging domains and their relationship to the epigenetic clock.

Regenerative Strategy	Targeted Aging Domain	Relationship to the Epigenetic Clock	Evidence
Cell therapy (MSC/TSPC/ADMSC, 3D bioprinting)	Progenitor competence	Biological age of donor/host cells governs potency	[40]
Secretome/exosomes (conditioned medium, EVs)	Senescence/SASP, angiogenesis	Readout of microenvironment reconditioning	[40]
Immunomodulation (MSC, M1→M2)	Inflammaging	Attenuates inflammaging components	[41]
Biomimetic gradient scaffold/patch (triphasic, bioprinted, ADM)	ECM/structure, morphogen presentation	Quantifies restoration of structural aging	[20,22,23,24,42,43]
Growth factor/morphogen (BMP/FGF-18/PRP)	Morphogen signaling	Supplies chondrogenic induction signals	[32]
Senotherapeutics (senolytics)	Senescence/SASP	Directly lowers clock components	[26]
Epigenetic reprogramming (partial OSK)	Upstream of all domains (cell age)	Resets the clock directly—readout and target	[44,45]

**Table 4:** Long-term scenarios that a validated tissue-specific epigenetic clock could ultimately enable: why each is not feasible today, and how such a clock could make it conceivable. None of these scenarios is supported by current clinical evidence; all are presented as long-term research goals rather than immediate perspectives.

New Possibility Opened by the Clock	Why It Is Impossible Today	How the Clock Makes It Possible
Rejuvenate-then-repair closed loop	There is no way to measure whether a preoperative intervention actually made the interface younger, so the strategy cannot be formulated.	Measure tissue age → apply a rejuvenation lever → re-measure → repair only once below a healing-competent threshold.
Molecular-age-timed surgery	Timing is set only by symptoms, structure (MRI), or chronological age; the irreversible aging threshold is unknown.	The clock signals a “biological deadline”, so surgery in performed before the interface crosses the point of no healing return.
Aging-signature-matched biologic	Which aging domain dominates is unknown, so PRP/cell therapy is applied unselected, with mixed results.	The clock profile distinguishes senescence- vs. inflammaging-driven deficits and routes to senolytic/anti-inflammatory/morphogenic levers.
Titrated in situ reprogramming	There is no readout to dose reprogramming without over-shooting into loss of identity or tumor risk.	A local clock provides a target rejuvenation set-point for closed-loop dose control that preserves cell identity.
Local–systemic targeting	Local enthesis aging cannot be separated from systemic aging, forcing reliance on systemic therapy.	If only the enthesis is old, a local biologic is used instead of a systemic drug (spatial targeting).
Clock-endpoint n-of-1/fast trials	Re-tear is a binary, months-later endpoint, so dozens of candidate biologics cannot be trialed efficiently.	A continuous, early clock endpoint enables small-sample, patient-as-own-control (n-of-1), and adaptive trials.

## Data Availability

No new data were created or analyzed in this study. Data sharing is not applicable to this article.
